# The impact of new psychosocial stresssors on the mental health of young people: Results from a national multicentric study in italy

**DOI:** 10.1192/j.eurpsy.2021.180

**Published:** 2021-08-13

**Authors:** G. Sampogna

**Affiliations:** Department Of Psychiatry, University of Campania “Luigi Vanvitelli”, Largo Madonna delle Grazie, Naples, Italy

**Keywords:** mental health, pandemic, Young People

## Abstract

The COVID-19 pandemic and its related containment measures, such as lockdown, is affecting mental health of the general population worldwide. This is an unprecedented event, which is influencing the health care, political, economic, and social welfare systems. Among Western countries, Italy has been one of the first severely hit by the pandemic in terms of number of cases and mortality rates. Therefore, on March 8, 2020, the Italian Prime Minister issued restrictive measures in order to limit the spread of the disease. During this period known as “Phase one” of the national health emergency, all not necessary activities have been closed, more than 29,000 people have died and almost 100,000 people have been home-isolated, with strict lockdown measures. The COvid Mental hEalth Trial (COMET) network, including ten university Italian sites and the National Institute of Health, has promoted a national online survey in order to evaluate the impact of lockdown measures on the mental health of the Italian general population. The COMET survey reports data from a large sample of more than 20.000 people from Italian general population, showing that lockdown has had a detrimental impact on mental health, in terms of worsening of anxiety, depressive and stress symptoms. Findings from this study can be useful to inform national and international associations, policy makers and stakeholders on the importance to provide adequate support to the mental health of the general population.

**Disclosure:**

No significant relationships.

